# Consumption of Spinach and Tomato Modifies Lipid Metabolism, Reducing Hepatic Steatosis in Rats

**DOI:** 10.3390/antiox9111041

**Published:** 2020-10-24

**Authors:** Laura Inés Elvira-Torales, Inmaculada Navarro-González, Joaquín Rodrigo-García, Juan Seva, Javier García-Alonso, María Jesús Periago-Castón

**Affiliations:** 1 Department of Food Technology, Food Science and Nutrition, Faculty of Veterinary Sciences, Regional Campus of International Excellence “Campus Mare Nostrum”, Biomedical Research Institute of Murcia (IMIB-Arrixaca-UMU), University Clinical Hospital “Virgen de la Arrixaca”, University of Murcia, Espinardo, 30071 Murcia, Spain; inmaculada.navarro@um.es (I.N.-G.); fjgarcia@um.es (J.G.-A.); 2 Department of Food Engineering, National Technological of Mexico, Tierra Blanca Campus, 95180 Tierra Blanca, Veracruz, Mexico; 3 Department of Health Sciences, Biomedical Sciences Institute, Autonomous University of Ciudad Juarez, 32310 Ciudad Juarez, Chihuahua, Mexico; jogarcia@uacj.mx; 4 Department of Anatomy and Comparative Pathological Anatomy, Faculty of Veterinary Sciences, Regional Campus of International Excellence “Campus Mare Nostrum”, University of Murcia, Espinardo, 30071 Murcia, Spain; jseva@um.es

**Keywords:** spinach, tomato, carotenoids, hepatic steatosis, lipid metabolism, gene expression, proteomic

## Abstract

Non-alcoholic fatty liver disease (NAFLD) is currently a serious and growing clinical problem in developed and developing countries and is considered one of the most frequent chronic liver diseases in the world. The aim of this study was to evaluate the functionality of dietary carotenoids provided by tomato and spinach in the dietary treatment of steatosis. Twenty-two Sprague-Dawley rats with induced steatosis were grouped into three groups and fed standard diet (CD group) and two experimental diets supplemented with 12.75% (LC12.75 group) and 25.5% (HC25.5 group) of a mixture of spinach and tomato powder. Rats fed carotenoid-rich feeds showed an improvement in the plasma biomarkers of steatosis, with lower levels of glucose, total cholesterol, VLDL, TG, proteins, ALT and AST. Likewise, a decrease in oxidative stress was observed, with a significant reduction of malondialdehyde (MDA) in plasma (up to 54%), liver (up to 51.42%) and urine (up to 78.89%) (*p* < 0.05) and an increase in plasma antioxidant capacity (ORAC) (up to 73.41%) (*p* < 0.05). Furthermore, carotenoid-rich diets led to an accumulation of carotenoids in the liver and were inversely correlated with the content of total cholesterol and hepatic triglycerides, increasing the concentrations of MUFA and PUFA (up to 32.6% and 48%, respectively) (*p* < 0.05). The accumulation of carotenoids in the liver caused the modulation of genes involved in lipid metabolism, and we particularly observed an overexpression of *ACOX1*, *APOA1* and *NRIH2* (*LXR*) and the synthesis of the proteins. This study suggests that dietary carotenoids from spinach and tomato aid in the dietary management of steatosis by reversing steatosis biomarkers.

## 1. Introduction

Among non-communicable diseases, chronic liver diseases (CLD) have become the new epidemic of this century, with non-alcoholic fatty liver disease (NAFLD) being one of the most important, associated with the increase in obesity and type 2 diabetes mellitus [1,2]. The prevalence of NAFLD is high on all continents; however, the highest rates are registered in South America (31%), the Middle East (32%), Asia (27%), the United States (24%) and Europe (23%), and it is less common in Africa [1]. NAFLD is the accumulation of excess fat in more than 5% of hepatocytes without significant alcohol intake [3]. Furthermore, this disease exhibits a histological spectrum, ranging from simple steatosis to the most aggressive necroinflammation, the so-called non-alcoholic steatohepatitis (NASH), which can progress to fibrosis, cirrhosis and ultimately hepatocellular carcinoma (HCC) [2,4]. In recent years, numerous studies have focused on the effect of the diet in the prevention of this disease, and there is unequivocal evidence that the consumption of carotenoids, ingested from plant foods, decreases the prevalence of NAFLD, mainly due to its potential to suppress reactive oxygen species and oxidative damage [5,6]. The incorporation of carotenoids in therapeutic diets against steatosis is of great interest since serum β-carotene levels in patients with NAFLD (with clinical signs of liver steatosis, inflammation and fibrosis) are significantly lower than in healthy subjects. Therefore, various authors have described that a higher consumption of this carotenoid in the diet from food sources is associated with a lower risk of primary liver cancer [7,8]. Vegetables such as spinach and tomato are a good source of dietary carotenoids, with values that can reach 10.87 and 7.82 mg/100 g of fresh weight, respectively [9]. Although carotenoids are recognized as antioxidants, the dietetic supplementation of carotenoids has not been used in the treatment of patients with NAFLD, but its beneficial effects have been studied experimentally using animal models [10,11,12,13,14,15,16].

Lutein protects against hepatic lipid accumulation and insulin resistance and attenuates lipid peroxidation by decreasing MDA (malondialdehyde) and the production of proinflammatory cytokines such as tumor necrosis factor-alpha (TNF-α) in the liver of guinea pigs. In addition, this antioxidant restored the expression of peroxisome proliferator-activated receptors (PPAR) that was inhibited by a high-fat diet supplied to rats, playing an important role in lipid metabolism [15,16]. The administration of zeaxanthin in gerbils from Mongolia reduces oxidative stress and liver fibrosis, associating this effect with its antioxidant capacity [17]. Eating foods high in β-carotene, such as goji berries, favors the modulation of the transcription factor NF-κB, the MAPK pathway and the autophagic process, also improving liver fibrosis, oxidative stress and the inflammatory response [18] in rats. In previous studies conducted in our research group, we observed that dietary supplementation with spinach in rats with induced steatosis leads to an accumulation of α- and β-carotene and lutein in the liver, showing an inverse correlation with total cholesterol and serum glucose and liver cholesterol content. Likewise, changes occurred in the expression of genes related to lipid metabolism, mainly through the overexpression of PPAR [14]. On the other hand, we found that lycopene-rich tomato juice is a therapeutic agent against NAFLD, since it reduces fat accumulation and inflammation in the liver, increasing the activity of mitochondrial and peroxisomal β-oxidation, the metabolite levels related to the antioxidant response, and modulating the expression of genes involved in lipid metabolism [10,11,12]. However, most of these findings related to the beneficial effect of carotenoids in NAFLD were investigated in animal models and were observed when dietary carotenoids were administered together with a high-fat diet. To our knowledge, few studies have been designed to evaluate the effect of carotenoids in the diet-mediated recuperation of steatosis. In addition, neither the synergistic effect of carotenoids from spinach and tomato nor their effect in the dietary treatment of the steatosis, after removing the high-fat diet, has been evaluated.

Taking into account the above, the objective of this study was to investigate whether spinach and tomato, and the carotenoids present in these foods, have effects on the diet-mediated recuperation of steatosis in rats. To achieve this goal, changes in plasma parameters, biomarkers of oxidative stress, lipid content in the liver and transcriptomic and proteomic profiles of rats with steatosis were evaluated after applying carotenoids-rich diets supplemented with tomato and spinach powder at different concentrations.

## 2. Materials and Methods 

### 2.1. Obtaining Spinach and Tomato and Preparation of Experimental Diets

Spinach (*Spinacia oleracea*) was purchased from a local supermarket as a fourth-range product (Florette SAS, Milagro, Navarra, Spain). The spinach was boiled for 10 min to reduce its oxalic acid content, the cooking water was discarded and the cooked spinach was lyophilized. The tomato paste was provided by a food company (Hero, Murcia, Spain) and subjected to lyophilization. Dry raw materials were crushed and stored at 4 °C. For the preparation of the experimental diets enriched with spinach and tomato, the standard powdered diet (Teklad Global 14%, Protein Rodent Maintenance Diet TD-2014, Harland Laboratories) was mixed with spinach and tomato powder in the following proportions 12.75% (7.12:5.63, respectively) and 25.5% (14.23:11.27, respectively). To the different mixtures of powder and feed, water was added to form a homogeneous mass to prepare the pellets, which were dried in an oven at 60 °C for 21 h. 

### 2.2. Chemical Composition of Experimental Diets

The proximal composition (moisture, ash, crude fat and total protein) was determined following the Association of Official Analytical Chemists (AOAC) [19] methods, and total carbohydrates were calculated by difference. The total dietary fiber content (TDF) was also determined following the method described by Prosky et al. [20]. Total phenolic compounds (TPC) were determined, using the Folin–Ciocalteu phenol reagent, according to Hirawan et al. [21].

### 2.3. Animals and Experimental Design

The experimental protocol of this study was approved by the Ethical Committee for Animal Experimentation of the University of Murcia and by the General Directorate of Livestock and Fisheries of the C.A.R.M. (No. A1320140701). Twenty-four adult male Sprague-Dawley rats (8 weeks old) were used, which were fed a high-fat diet (Atherogenic Rodent Diet TD-02028; Harland Laboratories) and a 20% fructose solution for 8 weeks with the goal of inducing steatosis [22,23]. After steatosis was confirmed by a histopathological study of the liver of two rats, the animals were classified into three experimental groups: control group (CD) (*n* = 6, standard diet), group with the low-carotene diet (LC12.75) (*n* = 8, standard diet supplemented with 12.75% mixture of spinach and tomato powder) and group with the high-carotene diet (HC25.5) (*n* = 8, standard diet supplemented with 25.5% mixture of spinach and tomato powder). The experimental period of administration of the carotenoid-rich diets was five weeks, as shown in the Supplementary Materials (Figure S1).

During the experimental period, a weekly body weight record was carried out, while food intake and urine and feces excretions for 24 h were recorded at the beginning and end of the experimentation period, using metabolic cages. After five weeks, and after a fasting period of 4 h, the animals were anesthetized with isoflurane, and blood extraction was performed by cardiac puncture. They were later sacrificed with an overdose of sodium pentobarbital by intraperitoneal injection (Dolethal, Laboratorios Vétoquinol, Spain). Heparinized tubes were used for the blood samples, and the plasma was obtained by centrifugation at 3000× *g* for 10 min at 4 °C. Subsequently, they were kept at −80 °C. At the same time, the other biological samples of feces, urine and liver were obtained, which were treated differently according to the destination that was to be given in subsequent analyses; they were stored at −80 °C until the moment of their use.

### 2.4. Histopathological Examination

Liver samples were taken from two rats at the end of the 8 weeks of steatosis induction to corroborate the mentioned pathology and also at the end of the experimental period to evaluate the evolution of histopathology during dietary treatment (*n* = 6). The rat liver samples were formalin-fixed and paraffin-embedded, and 4-µm-thick sections were obtained and stained with hematoxylin–eosin (H-E) for examination under an optical microscope. To determine the degree of steatosis, the percentage of hepatic cells with vacuolar degeneration was calculated in 10 fields (of 2600 µm^2^) in liver samples from each animal studied. The evaluation of steatosis was carried out according to the procedure described by Brunt et al. [24].

### 2.5. Plasma Biochemical Parameters

The concentrations of glucose, total cholesterol, HDL-cholesterol, LDL-cholesterol and VLDL-cholesterol, total triglycerides (TG) and total protein and the activity of the enzymes alanine and aspartate aminotransferase (ALT and AST) were analyzed using an automatic analyzer (A25 BioSystems SA, Barcelona, Spain) in the Animal Experimentation Service of the University of Murcia.

### 2.6. Determination of Biomarkers of Oxidative Stress

The total antioxidant capacity of plasma and diets, expressed as mmol of equivalents of Trolox (TE)/L, was measured using the oxygen radical absorption capacity (ORAC) technique, adapted for semi-automatic measurement in 96-well multimodal microplates (Synergy HT Biotek, Winooski, VT, USA) [25]. To assess the degree of lipid peroxidation, the levels of malondialdehyde (MDA) were analyzed in liver, plasma and urine samples by HPLC, following the method described by Mateos et al. [26]. Results were expressed as nmol MDA/g protein in the liver, nmol MDA/mL in plasma samples and nmol MDA/mg creatinine in urine. The protein concentration in the liver was analyzed following the Lowry method, modified by Bensadoun and Weinstein [27]. The creatinine concentration in urine was analyzed following the picric acid spectrophotometric method by the Jaffè reaction [28].

### 2.7. Carotenoid Analysis in Diets, Liver, Feces and Plasma 

Carotenoid analysis in experimental diets, liver and feces was performed according to the method described by Seybold et al. [29] with some modifications, as have been described in previous investigations [11,12,14]. The samples were extracted twice in tetrahydrofuran/methanol (1/1, *v*/*v*) with 0.1% of butylhydroxytoluene, and, after elimination of the solvent, the residues were reconstituted with a TBME/MeOH solution (1:1), centrifuged at 14,000 rpm for 10 min at room temperature and filtered. For plasma carotenoid analysis, it was performed according to the liquid–liquid extraction procedure previously described by Arranz et al. [30] with some modifications. Four hundred microliters of ethanol and 2 mL of hexane/BHT (0.1%) were mixed for 1 min and centrifuged at 2140× *g* for 5 min at 4 °C. The non-polar top layer was removed, and the remaining aqueous plasma mixture was re-extracted. The nonpolar aqueous extracts were evaporated with nitrogen gas at room temperature and reconstituted with 300 µL of TBME/MeOH. Carotenoids were analyzed by HPLC (Agilent 1200, Waldbronn, Germany) with a C30 column (250 × 4.6 mm, 5 μm id) (Trentec, Gerlinger, Germany) using the conditions described by Elvira-Torales et al. [14], using pure lycopene, lutein, zeaxanthin and β-carotene standards to quantify the carotenoid content.

### 2.8. Lipid Analysis in the Liver

For the determination of fatty acids and cholesterol, a Sigma-Aldrich lipid extraction kit (MAK174, St. Louis, MO, USA) and a cholesterol extraction kit (MAK175, St. Louis, MO, USA) were used, respectively, following the extraction method of Folch et al. [31]. The extracted supernatants were evaporated under flow of N_2_, reconstituted with hexane and immediately injected into a gas chromatograph (Agilent GC 7890A, Palo Alto, CA, USA) equipped with a flame ionization detector (FID), as reported by Martín-Pozuelo et al. [11]. Triglyceride analysis was performed using a triglyceride colorimetric assay kit (Cayman Chemical Company, Ann Arbor, MI, USA).

### 2.9. Study of the Expression of Genes Involved in Fatty Liver Metabolism

For gene expression analyses, liver samples were collected in sterile RNase-free microtubes with the addition of RNAlater^®^ solution in order to avoid RNA degradation during sample storage. RNA was extracted from the liver using a TNeasy^®^ mini kit (Quiagen, Duesseldorf, Germany) following the procedure described in the manufacturer’s instructions. After extraction, the quantity and quality of the RNA were evaluated using a Nano-Drop ND1000 (NanoDrop Technologies, Wilmington, NC, USA) and verified spectrophotometrically with ratios of 260/280 nm > 2.0 and ratios of 260/230 nm > 1.7. For reverse transcription, 1 µg of total RNA was used with an RT2 First Strand kit (Qiagen, SABioscience, Frederick, MD, USA). Reverse transcription was performed using a LightCycler 480 thermocycler (Roche Diagnostics Corporation, Indianapolis, IN, USA), and DNA samples were stored to carry out real-time PCR (qPCR) experiments with a specific array for the study of genes related to rat fatty liver disease (PARN-157ZD-24, Qiagen, SABiosciences, Frederick, MD, USA). The array has a 96-well plate designed for the evaluation of genes related to steatosis; however, in this research, we only included genes related to the β-oxidation of fatty acids and the transport and metabolism of cholesterol and other lipids. The obtained fluorescence results were analyzed using RT2 ProfilerTM PCR matrix data analysis, considering the control group as a reference to determine the relative expression change. The results were considered valid when a fold change was obtained in the gene expression ≥1.5 or ≤− 1.5, with statistically significant differences at *p* < 0.05, indicating an expression higher or lower than that found in the control group.

### 2.10. Western Blot 

For protein extraction, cell lysis was performed with a 50 mM Tris buffer, 1 mM EDTA, 150 mM NaCl, 5 mM MgCl_2_ and 0.5 mM 2-mercaptoethanol (98%) using a rotary homogenizer, followed by centrifugation of the lysates at 10,600 rpm for 5 min at 4 °C, and then the supernatant was collected. Proteins were quantified using the Lowry method, modified by Bensadoun and Weinstein [30]. Twenty micrograms of rat liver proteins were separated by 12% SDS–polyacrylamide gel electrophoresis and transferred to PVDF membranes. Immediately afterwards, the membranes were blocked with 3% bovine serum albumin (BSA) in TBS (50 mM Tris, pH 7.5, 0.15 M NaCl) for 2 h at 4 °C and incubated for 12 h at 4 °C with primary anti-ACOX1 antibodies (abcam, ab184032; 1:1000), anti-LXR-β (abcam, ab28479; 1:1000), anti-apoA1 (abcam, ab20453; 1:1000) and anti-F-actin (abcam, ab205; 1:500). The membranes were washed three times (5 min each) with T-TBS solution (0.1% Tween-20 in TBS) and immediately incubated for 2 h at 25 °C with an anti-mouse HRP-conjugated secondary antibody (PIERCE, 1858413; 1:100,000) or a secondary anti-rabbit HRP-conjugated antibody (PIERCE, 1858415; 1:100,000). Finally, the membranes were washed three times with T-TBS and then 2 times with TBS. Signals were detected with chemiluminescent HRP substrate using the SuperSignal West Femto Maximum Sensitivity Substrate Kit (Thermo Scientific, Rockford, IL, USA). To obtain the images of the bands, the chemiDoc-It2 UVP imaging system (UVP, LLC, Upland, CA, USA) was used. The intensity of the bands in the images was determined using Image Studio Lite 5.2 software (LI-COR Biosciences, Lincoln, NE, USA). The reported values were normalized with F-actin. To verify the processed proteins, the molecular weight was determined by contrasting with the standard.

### 2.11. Statistical Analysis

All experiments were performed in triplicate. In all cases, the normality and equality of variances were tested. The normal distribution of the data was verified with the Ryan Joiner normality test (similar to Shapiro–Wilk), and the test of equal variances was checked by Bartlett’s test. The data obtained from this investigation were analyzed using a one-way ANOVA with repeated means, with a post-hoc test to check the differences between the averages of all the analytical determinations, the Tukey test or the Games–Howell test, as appropriate. Furthermore, for the parameter of MDA in urine, the paired Student’s *t*-test was performed to compare the values at the beginning and at the end of the experiment. The data were expressed as mean values ± SD of the results obtained. The level of significance was *p* < 0.05. The Minitab statistical software package version 17.0 (Minitab, LLC., State College, PA, USA) was used. Analysis of differentially expressed genes was performed with Partek Genomics Suite 6.6.3. 

## 3. Results

### 3.1. Daily Intake of Feed and Biometric Parameters

As can be seen in Table S1, the proximal composition, energy values, antioxidant capacity (measured by the ORAC method) and carotenoids of the diets supplemented with spinach and tomato showed significant differences among the three experimental diets. In rats fed the diet high in carotenoids (HC25.5), compared with rats of the CD group, the intake of protein, fat and dietary fiber was significantly higher (1.13, 1.17 and 1.21 times, respectively) and the carbohydrate consumption was lower (1.15 times lower), resulting in a decrease in energy value (306.59 kcal/100 g). Although this caloric difference was significant in absolute value, the diet of the HC25.5 group was only 5% lower in total caloric value than the standard one, showing the same amounts of calories from carbohydrates. A similar trend was observed in the low carotenoid group (LC12.75); however, it does not show statistically significant differences with the control group for fiber and carbohydrate intake and with the HC25.5 group for content protein and fat.

As expected, supplementation with the mixture of spinach and tomato provided a significant increase in the content of total phenolic compounds, carotenoids and ORAC, which increased significantly according to the percentage of lyophilized vegetables included in the diet. For phenolic compounds, the mean values ranged from 205.03 to 265.30 mg/100 g for the CD and HC25.5 diets, respectively. The experimental diets showed a wide range of carotenoids, among which neoxanthin, violaxanthin, lutein, zeaxanthin, phytoene, α-carotene, β-carotene and lycopene stand out. The carotenoids content ranged from 283.84 to 528.65 μg/g, for HC25.5 and LC12.75 diets, respectively, while carotenoids were not detected in the CD. Regarding the ORAC, a significant increase was observed in concordance with the addition of plant foods. 

Table 1 shows food and water consumption, changes in body weight, excretion of feces and urine, intake of phenolic compounds and total dietary fiber (TDF) and absorption of carotenoids during the experimental period. No significant differences were found in the initial and final weights between the experimental groups, due to the variability found in the weights of the different animals. However, the body weight decreased 27.5 and 50.17 g during the study in the LC12.75 and HC25.5 groups, respectively, whereas, in the CD groups, it remained almost unchanged. This fact was determined by a slight decrease in the caloric value of the experimental diets compared to the standard maintenance diet administered to the control group. Regarding the behavior of the animals and the acceptance of the diet, no significant changes were observed for the intake of food and water or the excretion of urine and feces among the three experimental groups.

The intake of carotenoids was significantly higher in the HC25.5 group (8.07 mg/day) than in the LC12.75 group (4 mg/day). Considering the amount of carotenoids excreted in feces, the apparent absorption was calculated, which presented the same trend, namely 54.94% and 69.02% in the LC12.75 and HC25.5 groups, respectively. Taking into account that plant foods are a natural source of dietary fiber, the daily intake of TDF was also calculated, but there were no significant differences among the three experimental groups.

### 3.2. Histopathological Examination and Biochemical Parameters 

At the beginning of the five-week intervention period and according to the histopathological examination, the animals had developed steatosis, showing vacuolar degeneration of the hepatocytes (images not shown) [27]. Furthermore, diet-induced steatosis was corroborated by the activity of liver enzymes alanine aminotransferase (ALT) and aspartate aminotransferase (AST), whose activities were greatly increased (84.83 U/L and 161.30 U/L, respectively), as well as by the increase in plasma triglycerides (189 mg/dL) and the high level of MDA in urine (19 nmol MDA/mg creatinine) (data not shown).

**Figure 1:**
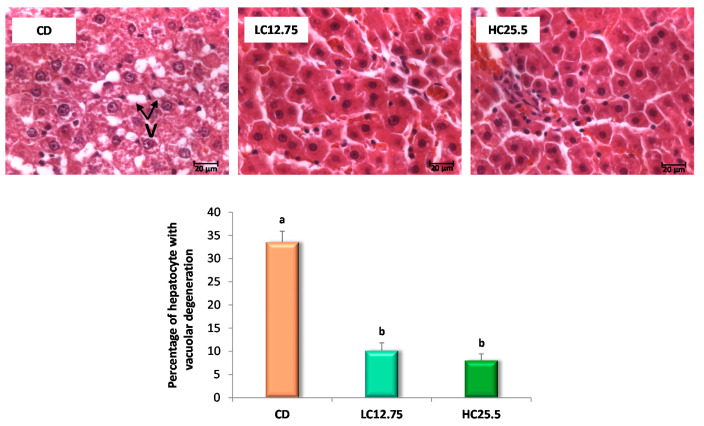
Microscopic images of liver with H-E visualized by optical microscopy (63×) for the three experimental groups (control group, CD; low in carotenes, LC12.75; high in carotenes, HC25.5) at the end of the five-week intervention period. The arrows show the vacuolar degeneration of the hepatocyte (V). The bar diagram shows the percentage of hepatocytes with vacuolar degeneration. Data are expressed as mean value and SD, the percentage of hepatic cells with vacuolar degeneration was calculated in 10 fields (of 2600 µm^2^ for each group (*n* = 6 for CD group, *n* = 6 for LC12.75 and HC25.5 groups). ^a,b^ Different letters show significant statistical differences between the groups after one-way ANOVA (*p* < 0.05).

**Figure 2:**
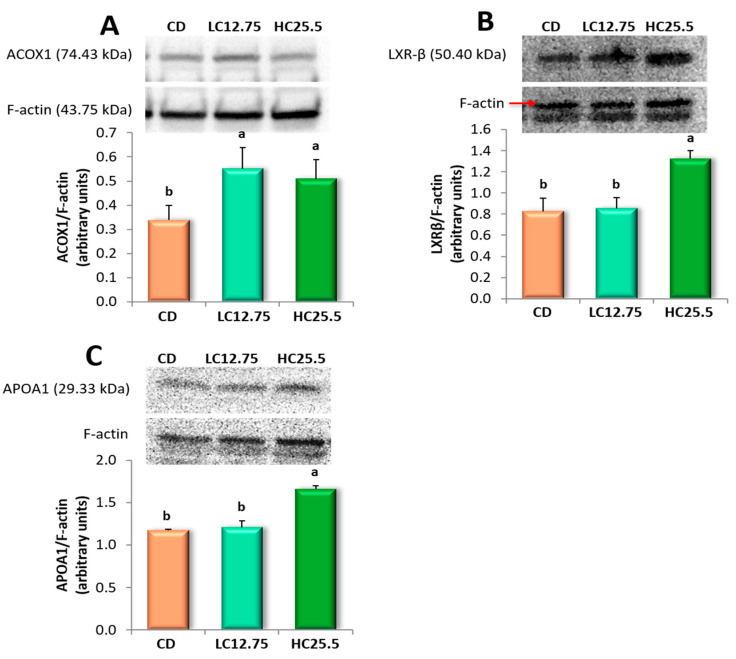
Effect of dietary supplementation of spinach and tomato mixture on protein levels measured by Western blot in rat liver samples of: (**A**) ACOX1; (**B**) LXR-β; and (**C**) APOA1. The representative image of the triplicate experiments is shown in the top panel. The bottom panel shows the intensity of the bands measured densitometrically and normalized to the expression levels of F-actin protein. Data are expressed as mean value for each group (*n* = 6 for DC group, *n* = 8 for LC12.75 and HC25.5 groups) analyzed individually in triplicate ± SD. ^a,b^ Different letters show significant statistical differences between the groups after one-way ANOVA *(p* < 0.05).

The histopathological examination was also performed at the end of the five-week intervention period with the carotenoids-rich diets. Rats from the CD group had Grade 2 steatosis (more than 30% of hepatocytes with vacuolar degeneration) and a low grade of inflammation (Grade 1, small number of inflammatory foci); however, the rats that ingested the diets supplemented with spinach and tomato (LC12.75 and HC25.5) showed a low degree of steatosis (Grade 1), with a lesser percentage of hepatocytes affected (Figure 1). Furthermore, in these last two groups (LC12.75 and HC25.5), significantly smaller vacuoles were observed (Figure 2). These results show a better state of the liver after carotenoid intervention, which was accompanied by a significant decrease in liver enzymes. Thus, the activity of the ALT enzyme was reduced 1.4 and 1.6 times, while the AST enzyme 1.7 and 1.9 times, in the LC12.75 and HC25.5 groups, respectively, compared to the CD group (Table 2).

Plasma biochemical parameters, plasma lycopene concentrations and parameters related to the oxidative metabolism and oxidative stress are shown in Table 2. At the end of the experiment, significantly lower levels of glucose, proteins, total cholesterol, VLDL-cholesterol and triglycerides were shown in the groups fed the mixture of spinach and tomato. For total cholesterol, total triglycerides and VLDL, the decrease was directly proportional to the carotenoids content in the diet, indicating that a higher intake may have a greater effect on these plasma parameters. However, no significant differences were observed in the HDL-cholesterol and LDL-cholesterol fractions between the three experimental groups. In general, animals from the CD groups showed the highest levels of glucose, total cholesterol, triglycerides and ALT and AST enzymes, which are due to the metabolic disorders clearly related to the steatosis.

Lycopene was the only carotenoid detected in plasma, ranging from 0.013 to 0.016 μg/mL for the LC12.75 and HC25.5 groups, respectively, and showing significant differences. The intake of diets rich in carotenoids significantly increased the ORAC in plasma from 7.56 mmol TE/L in the CD group to 10.40 and 13.11 mmol TE/L in the experimental groups. This behavior was proportional to the intake carotenoids, representing an increase of 37% and 73% in LC12.75 and HC25.5 groups, respectively, compared with CD. The MDA levels in plasma, liver and urine were significantly higher in the CD group in comparison with the other two experimental groups, indicating an increase in lipid peroxidation as a result of lipid accumulation in the liver due to the steatosis. On the contrary, the concentration of MDA was significantly reduced in plasma, liver and urine samples of rats with the carotenoid-rich diets.

The MDA values detected in the urine samples at the beginning of the intervention study showed mean values around 19 nmol MDA/mg creatinine for the rats of the three groups, due to the intake of the high-fat diet (data not shown). After five weeks of intervention with diets rich in carotenoids, a significant decrease was observed in urine MDA values. Although rats of the CD group showed a significant decrease of the initial MDA values (18.55 vs. 14.07 nmol MDA/mg), this reduction was around 25%, whereas, in LC12.75 and HC25.5, it was around 70% and 85%, respectively (19.79 and 19.20 vs. 6.20 and 2.97 nmol MDA/mg). This tendency revealed that dietary intake of carotenoids contributes efficiently to the reduction of lipid oxidation biomarkers associated with steatosis, improving this hallmark. 

### 3.3. Carotenoid Content in the Liver

The total content of carotenoids in the liver showed a significant difference between the two groups that consumed the mixture of spinach and tomato (Table 3), with mean values of 589.21 and 1043.84 μg/g in the LC12.75 and HC25.5 groups, respectively. For all individual carotenoids, rats from the HC25.5 group also showed a significantly higher content than rats from the LC12.75 group. The highest concentration was for lycopene, followed by phytoene, lutein, β-carotene, α-carotene, zeaxanthin, neoxanthin and violaxanthin. On the contrary, carotenoids were not detected in the liver of rats from the CD group.

### 3.4. Total Fat, Cholesterol, Triglyceride and Fatty Acid Content in the Liver

The lipid profile of the liver was analyzed in liver samples (Table 4). Although the total fat content of the liver only showed significant differences for rats from HC25.5, total cholesterol and triglycerides concentrations decreased significantly after ingestion of the diet supplemented with spinach and tomato, decreasing by 41% and 28%, respectively, in the LC12.75 group and 51% and 46%, respectively, in the HC25.5 group, compared to the mean values of the CD group. Considering that the main difference in the composition of the diets was the carotenoid content, and since the intake of the nutrients, including fat, only varied by no more than 0.5% in the experimental diets, these results suggest that the supplementation of the diet with spinach and tomato and the accumulation of carotenoids in the liver have a positive effect on lipid metabolism in the liver, reducing total fat, total cholesterol and triglycerides. Moreover, differences in the amounts of individual fatty acids and in the fatty acid profile were observed, indicating differences in the qualitative and quantitative accumulation of fat. With regard to the concentration, it is important to note the significant increase in n-3 (linolenic acid (ALA), eicosapentaenoic acid (EPA) and docosahexaenoic acid (DHA)) and n-6 (linoleic acid (LA), eicosadienoic acid (EDA) and arachidonic fatty acid (AA)) in animals fed with the higher concentration of spinach and tomato. For this reason, the incorporation of these carotenoid-rich vegetables positively influenced the composition of fatty acids, significantly reducing the content of saturated fatty acids (SAFA) (24.49% in LC12.75 and 18.50% in HC25.5 vs. 41.16% in CD) and significantly increasing the content of monounsaturated fatty acids (MUFA) (32.67% in HC25.5 vs. 24.94% in CD) and polyunsaturated fatty acids (PUFA) (around 48% in LC12.75 and HC25.5 compared to 33.9% in CD). In addition, a significant decrease in the n-6/n-3 ratio was observed in the HC25.5 group.

### 3.5. Identification of the Genes Related to NAFLD

Table 5 shows the nine genes of lipid metabolism differentially expressed (*p* < 0.05) with a fold change greater than ± 1.5 for the rats of experimental groups LC12.75 and HC25.5, considering the CD as a reference group. All the genes related to lipid metabolism showed a differential expression revealing an overexpression of mRNA, showing a positive fold change. Changes in relative expression in groups LC12.75 and HC25.5 showed that diets supplemented with spinach and tomato modified the liver transcriptome. Thus, in the HC25.5 group, a significant overexpression was observed for genes related to β-oxidation (*ACOX1*), cholesterol metabolism and transport (*APOA1*, *CNBP*, *NR1H2* and *PPARD*), while for the animals of the LC12.75 group, two genes related to β-oxidation (*ACOX1* and *ACADL*) were overexpressed, as well as the *CYP2E1* gene, related to cholesterol metabolism and transport, and *ACSM3* and *HNF4A* genes, involved in metabolism and transport of other lipids. 

### 3.6. ACOX1, LXRβ and APOA1 Protein Expressions in the Liver

We selected three genes to assess whether the overexpression was correlated with increased protein synthesis (Figure 2). Thus, Western blot analysis of the liver tissues of rats fed the supplemented diet (LC12.75 and HC25.5) revealed that the expression of the ACOX1 protein increased significantly in the two groups, while the level of LXRβ and APOA1 proteins alone increased significantly in the livers of rats fed a higher percentage of carotenoids (HC25.5) compared to rats fed CD. Therefore, the supplementation of the feed with the mixture of spinach and tomato led to an increase in the synthesis of ACOX1 (CD, 1.0 vs. 1.62 and 1.50, in LC12.75 and HC25.5, respectively), LXRβ (2.43 in CD vs. 3.90 in HC25.5) and APOA1 (3.45 in CD, vs. 4.88 in HC25.5) compared to the CD group.

## 4. Discussion

### 4.1. Carotenoid Intake and its Action on Steatosis Biomarkers 

Several authors have pointed out that dietary fat, cholesterol and fructose are the main drivers of the development and progression of obesity and steatosis [22,23]. In the present study, an atherogenic (21% fat and 1.25% cholesterol) and high-fructose (20%) diet was used for the induction of hepatic steatosis for eight weeks. The disease was confirmed by histopathological analysis of the liver, increased plasma triglycerides and the activity of liver enzymes in plasma, which were outside the range considered normal [32]. Taking into consideration the carotenoid intake and the average weight of the rats (0.5 kg), the ingested dose of carotenoids was 8 and 16 mg/kg/day in LC12.75 and HC25.5, respectively, and these values are within those that have been considered safe in different toxicity studies. Specifically, the Panel of the European Food Safety Authority (EFSA), after evaluating a study carried out with rats, has considered a maximum dose with no observed adverse effect level (NOAEL) of 500 mg/kg/day for lycopene, 538 mg/kg/day for lutein and 150 mg/kg/day for zeaxanthin, since, although they accumulate in the liver, the presence of pigments without the existence of histopathological alterations is not relevant from a toxicological point of view [33,34].

Firstly, supplementation of the feed with tomato and spinach concentrates was sufficient to significantly reduce the bodyweight of the rats by 5% and 8%, respectively. In overweight people at metabolic risk, a 5% reduction in body weight has been shown to reduce the incidence of type 2 diabetes by 58% [35]; hence, a relatively moderate weight loss is capable of causing improvements in metabolic control. This fact directly affects the treatment of NAFLD, since overweight and diabetes play a fundamental role in the appearance of the disease [36,37]. This effect could be associated with lower caloric intake, but other factors in the experimental diets may also influence weight control, such as the higher consumption of TDF. Parallel to the changes observed in body weight, supplementation with the mixture of spinach and tomato improved the lipid profile of the plasma, notably reducing the levels of glucose, total cholesterol, VLDL, triglycerides, proteins, ALT and AST compared to the control group. This lipid-lowering effect of carotenoids has been described in other previous studies in obese animals with NAFLD [38,39] and is due to different mechanisms. Thus, lycopene has been shown to have a hypocholesterolemic effect, as it is capable of inhibiting the cholesterol synthesis limiting enzyme 3-hydroxy-3-methylglutaryl-CoA reductase (HMGCR) [40,41], although it is also capable of modulating the expression of genes involved in lipid metabolism, mainly due to the modulation of genes involved in the signaling pathways of PPARs [10,11,12,14]. The lipid-lowering and hypocholesterolemic effects are directly related to the accumulation of lycopene after ingestion, since this was the majority carotenoid in the experimental diets and the only carotenoid found at the plasma level, with concentrations ranging from 13 to 15 ng/mL, as a function of lycopene concentration in the diets. These values coincide with the lycopene levels reported by Luvizotto et al. [42], who found a mean value of 12.67 ng of lycopene/mL after consumption of 3.5 mg of this carotenoid by Wistar rats. The ORAC value represents an adequate biochemical parameter to assess the general status of antioxidants [25,43], and it increased after the consumption of a mixture of spinach and tomato in the diet, since, parallel to the increase in lycopene, ORAC in plasma was increased. 

Although carotenoids other than lycopene were not detected in the plasma, the carotenoids present in the experimental feeds were accumulated in the liver, with the HC25.5 group showing the highest content. Therefore, the different carotenoids, namely lutein, zeaxanthin, phytoene, β-carotene and lycopene, were absorbed and accumulated in the liver to later be incorporated into the lipoproteins for release into the circulation [44]. In the liver, these micronutrients exert a clear antioxidant effect, acting effectively against free radical species, which results in less lipoperoxidation and a significant decrease in MDA content. This effect was clearly observed in the LC12.75 and HC25.5 groups, since MDA levels were significantly lower than in the control group, and, in the latter, a greater accumulation of MDA as a result of excessive lipid peroxidation was observed [45]. This fact is also reflected in other oxidative stress biomarkers such as MDA content in plasma and urine. These data coincide with those reported by other researchers, who observed that some carotenoids such as lycopene, β-carotene and lutein decrease MDA in plasma and liver of rats and guinea pigs with steatosis and NASH induced by a hypercholesterolemic diet or by carbon tetrachloride (CCL_4_) [15,39,46]. Thus, carotenoids play an important role in defense against oxidative stress by preventing or delaying oxidation through the neutralization of free radicals by sequestering singlet oxygen [47]. Carotenoids are considered beneficial for the prevention and treatment of NAFLD due to the role they play in reducing oxidative damage [48] in the diet-mediated recuperation of steatosis. 

Although carotenoids were absorbed and accumulated in the liver, a fecal analysis determined that 100% of the ingested carotenoids were not absorbed and were excreted in the feces, as has been described by other authors [49,50]. Although the maximum absorption of these bioactive compounds is always sought, there are many factors that can affect their intestinal absorption, thus it must be taken into account that a proportion of them will reach the colon, where they can affect the intestinal microbiota or even act as antioxidants for colon cells [51].

Recent studies in animal models indicate that the accumulation of fat and cholesterol in the hepatocyte are key factors for the activation of the inflammatory pathways involved in the transition from NAFLD to NASH and to hepatocellular carcinoma in animal models [52]. The intake of carotenoid-rich diets (groups LC12.75 and HC25.5) decreased the concentrations of total cholesterol and liver triglycerides, as has been found in other investigations with the supplementation of lutein, lycopene, astaxanthin and β-carotene [14,16,53,54]. The livers of the rats from the LC12.75 and HC25.5 groups, in addition to having a lower steatosis score than that of the CD group, showed less cholesterol accumulation, possibly due to its mobilization by plasma HDL [52,53]. This could explain the decrease in the concentration of cholesterol in the liver. Furthermore, as previously mentioned, other mechanisms that could contribute to the reduction of cholesterol in the liver are the hypocholesterolemic effect of lycopene (majority carotenoid accumulated in the liver), which inhibits the activity of HMGCR; the regulation of gene expression involved in lipid metabolism [11,40,41]; and an increase in the bile acid secretion [55]. However, this last mechanism was not evaluated in this study since we did not analyze the content of bile acids in the feces. It is worth mentioning that, even though a complete recovery from steatosis was not observed in the microscopic study, the differences in the size of the vacuoles associated with the intake of carotenoids were evident. Likewise, the intake of dietary carotenoids showed a positive effect by effectively decreasing the levels of total cholesterol and liver triglycerides, decreasing lipid accumulation and helping to restore liver function.

MUFAs and PUFAs increased while SAFAs and the ratio of n-6/n-3 decreased after the intake of carotenoid-rich diets. MUFAs and PUFAs exert a beneficial effect on the liver, modulating the activity of liver cells during liver fibrosis [56]. This increase in MUFAs can be beneficial for liver health, since different authors have shown that MUFAs can improve insulin resistance, increase the release of triglycerides in the liver, decrease lipolytic flow of peripheral adipose tissue [57,58] and inhibit kinases related to oxidative stress and apoptosis [59] in human and animal models with NAFLD. Moreover, the increase in the proportion of PUFAs suggests that the accumulation of hepatic carotenoids stimulates the conversion of fatty acids into long-chain unsaturated products, as mentioned in the previous study with the supplementation of spinach in the diet [14]. The production of ALA (C18:3n-3 α), DGLA (C20:3n-3), EPA (C20: 5n-3) and DHA (C22:6n-3) was increased in the LC12.75 and HC25.5 groups. In general, n-3 AGPs have been shown to have effects against obesity, steatosis and inflammation [60], as well as glucose and lipid metabolism [61]. These effects could be due to the stimulation of fat catabolism and the inhibition of de novo fatty acid synthesis [57]. In our study, the increase in n-3 fatty acids was significantly correlated with the accumulation of carotenoids and the decrease of lipids in the liver. Furthermore, the decrease in the n-6/n-3 ratio has an effect on the reduction of hepatic triglyceride storage [57], as observed in the present study, contributing carotenoids in the diet-mediated recuperation of steatosis. 

### 4.2. Bioactive Components of Spinach and Tomato Modulate Gene Expression Related to Steatosis

**Figure 3:**
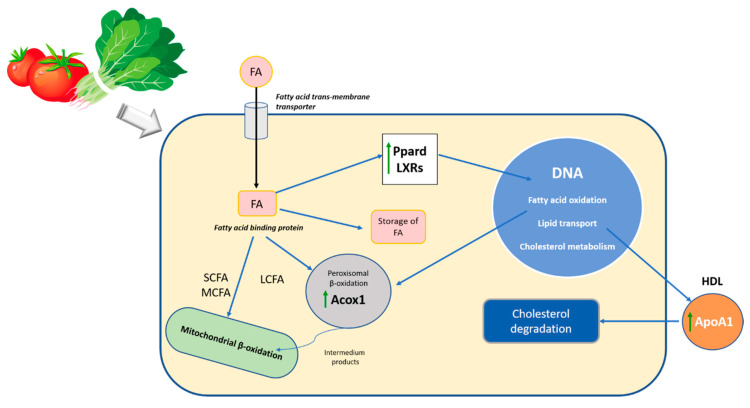
Schematic representation of the effect of carotenoids in the lipid metabolism during the diet-mediated recuperation of steatosis. FA, fatty acids; SCFA, short-chain fatty acids; MCFA, medium-chain fatty acids; LCFA, long-chain fatty acids; Ppard, peroxisome proliferator-activated receptor delta; LXRs, Acox1, Acyl-Coenzyme A oxidase 1; palmitoyl; ApoA1, Apolipoprotein A-1; DNA, deoxyribonucleic acid; HDL, high-density lipoprotein.

In this study, we observed that the ingestion of a diet rich in carotenoids after the induction of steatosis and during its dietetic treatment leads to changes in the expression of genes related to fatty liver disease. The main carotenoids of the diets, lycopene and lutein, are transported into the hepatocyte by the transmembrane transporter and by fatty acid-binding protein into the cytosol, where carotenoids act as activators of different nuclear receptors, which are involved in the transcriptional regulation of several pathways of lipid metabolism [62] (Figure 3). 

The overexpression of the *ACOX1* gene, as well as the synthesis of peroxisomal ACOX1 protein, could indicate a healthier status to maintain the peroxisome activity of β-oxidation. Thus, these findings suggest that the accumulation of carotenoids in the liver favors the peroxisomal lipid oxidation by improving the breakdown of medium and very long-chain fatty acids. Consequently, the carotenoids could aid in the recuperation of the homeostasis of liver lipids, as has been reported in other studies [54,63]. In fact, we observed a significant decrease in cholesterol, TGA and SAFA in the liver, decreasing the lipid peroxidation and protecting the liver against inflammation and fibrosis. It is recognized that this protein plays an important role in the NAFLD, as ACOX1-deficient mice have shown spontaneous steatosis and steatohepatitis, as well as the spontaneous development of hepatocellular carcinoma (HCC) [64]. Similarly, it has been observed that patients with pseudoneonatal adrenoleukodystrophy (a disease characterized by ACOX1 deficiency) show hepatomegaly due to impaired peroxisomal fatty acid β-oxidation of very long-chain fatty acids [65]. *ACADL,* which encodes a dehydrogenase enzyme that catalyzes the initial step in each cycle of mitochondrial fatty acid β-oxidation [66], was also overexpressed in the group LC12.75; this effect was also described after the consumption of tomato juice in rats with steatosis [11].

Furthermore, the genes involved in cholesterol transport and metabolism increased their activity, overexpressing *APOA1*, *CNBP*, *NR1H2* and *PPARD* in the HC25.5 group. The *APOA1* gene encodes apolipoprotein A-1, which is the main protein of HDL in plasma, which defines its size and shape, solubilizes its lipid component and helps to reverse cholesterol transport [67]. It also acts as a cofactor of lecithin cholesterol acyltransferase (LCAT) for the formation of most cholesterol esters in plasma, promoting the release of cholesterol from the tissues to the liver for its subsequent excretion [68]. Recent studies indicate that metabolic alterations in the body, including changes in the levels and quality of APOA1, may favor the appearance and progression of some types of cancer, such as HCC, showing that the development of hepatocellular carcinoma is associated with lower levels of apolipoprotein A-1 [69,70,71]. The findings of this study indicate that a greater expression of the APOA1 protein is proportional to the carotenoids ingested in the diet. The high consumption of carotenoids could promote atheroprotective properties, since the overexpression of *APOA1* could improve the reverse cholesterol transport, decreasing the cholesterol content in the liver and improving the hallmarks of the steatosis and then the progression of NAFLD [72]. *CNBP* was also overexpressed in the liver after the consumption of a diet with high carotenoid content. This gene is expressed in a wide variety of tissues, is regulated by sterols and plays an important role in the control of sterol-mediated transcription [73]. Similarly, rats from the HC25.5 group showed an expression of the *LXRB* gene (*NR1H2*) and the synthesis of its protein (LXRβ). These lipid-activated transcription factors heterodimerize with the Retinoid X Receptor (RXR) to control cholesterol and fatty acid homeostasis by regulating the expression of multiple enzymes, transporters and modulators involved in this process and also by modulating inflammatory and immune pathways. Hence, LXRs have been proposed as effective therapeutic agents against NAFLD [74]. Recently, Becares et al. [75] reported that LXRs phosphorylation acts as a molecular sensor in response to nutritional challenges, thus promoting a diet-induced transcriptome that modulates metabolic and inflammatory responses in NAFLD progression. Therefore, accumulation of carotenoids could play an important role in the phosphorylation of these transcription factors, leading to a decrease in lipids accumulation on the hepatocyte and of oxidative stress biomarkers.

The ingestion of spinach and tomato modulated lipid metabolism in the LC12.75 group, overexpressing the *ACSM3* gene, which encodes a protein that participates in the synthesis of medium-chain fatty acids, and showing an increase in lipid metabolism. As mentioned in a previous study, the activity of this encoded protein facilitates the oxidation of medium-chain fatty acids (C4–C11); for this reason, rats fed carotenoids had a lower SAFA/TFA ratio at the liver level [14], as can be seen in Table 4. The *HNF4A* gene encodes the nuclear transcription factor protein that binds to DNA, controlling the expression of several liver genes (nuclear hepatocyte factor 1 alpha), also regulating the genes involved in the metabolism of cholesterol, fatty acids and glucose [76,77].

In this study, we confirmed that the consumption of carotenoids from spinach and tomato facilitates the recuperation of steatosis, and that the accumulation of carotenoids in the liver also seems to have a protective effect on the diet-mediated recuperation of steatosis. The ameliorative effect is mediated through the modulation of genes involved in lipid metabolism, especially by improving the β-oxidation of peroxisomal fatty acids through ACOX1, improving the homeostasis of cholesterol and fatty acids through LXRB and promoting the release of cholesterol from tissues to the liver for subsequent excretion thanks to APOA1. Therefore, our findings provide a new perspective on the nutritional value of dietary carotenoids from tomato and spinach in the treatment of NAFLD.

The present study describes the influence of the synergistic effect of two foods rich in carotenoids (neoaxanthin, violaxanthin, lutein, zeaxanthin, phytoene, α-carotene, β-carotene and lycopene) with two different concentrations of these bioactive compounds in the diet. This research was carried out with experimental animals (Sprague-Dawley rats) in which hepatic steatosis was previously induced. Our results provide additional evidence of the synergistic and potential effect of dietary carotenoids on liver health and dietary treatment of steatosis, suggesting that the carotenoids provided by the spinach and tomato are effective in reducing the accumulation of lipids in the liver, oxidative stress and inflammation of hepatocytes and increasing the body’s antioxidant activity, modulating the intracellular signaling pathways of the β-oxidation of fatty acids and the metabolism of lipids and cholesterol at the genomic and proteomic level.

A limitation of the present study is that, as it was carried out with animals, it cannot be completely extrapolated to humans, so a clinical study is needed in both healthy people without diseases and those with steatosis problems due to excessive consumption of fats and carbohydrates. Such a study would aim to corroborate the protective function of dietary carotenoids as well as evaluate whether there could be an effective dose, considering the bioavailability of dietary carotenoids, for the prevention of NAFLD.

## 5. Conclusions

The present work illustrates that the administration of spinach and tomato as a source of dietary carotenoids improves the hallmarks of steatosis by reducing the total cholesterol, triglycerides and SAFA, increasing MUFA and PUFA in the liver and improving the antioxidant status associated with the steatosis by reducing lipid peroxidation and increasing the antioxidant capacity in plasma. These changes are due to the antioxidant effect of carotenoids and are also partially explained by the modulation of genes related to lipid metabolism and other metabolic pathways of fatty liver disease, particularly through the overexpression of *ACOX1*, *APOA1* and *NR1H2* (*LXR*) and the synthesis of these proteins. These findings suggest that the supplementation of the diet with carotenoids could be a good strategy in the dietary treatment of steatosis. Hence, carotenoid-rich vegetables should be selected in the therapeutic diets for the recuperation of steatosis.

## Figures and Tables

**Table 1:** Food and drink intakes, feces and urine excretion and carotenoid consumption in the three experimental groups during the five-week intervention period ^1^.

Parameters	CD	LC12.75	HC25.5
Initial body weight (g)	554 ± 63.7	547 ± 68.4	546 ± 82.5
Final body weight (g)	551 ± 64.1	523 ± 49.2	506 ± 60.9
Body weight decrease (g)	2.83 ± 1.94 ^b^	27.5 ± 10.5 ^a^	50.1 ± 23.6 ^a^
Food intake (g/day)	14.7 ± 4.81	15.0 ± 5.11	15.2 ± 3.23
Water intake (mL/day)	32.4 ± 5.70	31.6 ± 1.43	32.4 ± 8.66
Excreted feces (g/day)	2.46 ± 0.69	3.05 ± 0.82	3.49 ± 0.92
Excreted urine (mL/day)	14.6 ± 1.13	13.9 ± 2.62	16.1 ± 5.4
Carotenoid intake (mg/day)	-	4.00 ± 1.35 ^b^	8.07 ± 1.71 ^a^
Carotenoids excreted in feces (mg/day)	-	1.80 ± 0.48 ^b^	3.64 ± 0.96 ^a^
Apparent carotenoids absorption (%)	-	54.9 ± 14.1	69.0 ± 13.3
TDF intake (g/day)	3.11 ± 1.02	3.21 ± 1.09	3.67 ± 1.03
TPC intake (mg GAE/day)	32.8 ± 9.8	36.7 ± 12.4	37.9 ± 10.6

^1^ Data are expressed as mean value for each group (*n* = 6 for DC group, *n* = 8 for LC12.75 and HC25.5 groups) analyzed individually in triplicate ± SD. ^a,b^ Different letters show significant statistical differences between the groups after performing a one-way ANOVA (*p* < 0.05). CD, Standard diet; LC12.75, Standard diet + 12.75% spinach and tomato mixture; HC25.5, Standard diet + 25.5% spinach and tomato mixture.

**Table 2:** Plasma biochemical parameters, liver enzyme activities, plasma lycopene concentration and oxidative stress biomarkers in different biological samples for the three experimental groups after the 5-week intervention period ^1^.

Parameters	CD	LC12.75	HC25.5
Glucose (mg/dL)	205 ± 15.5 ^a^	156 ± 26.2 ^b^	140 ± 23.7 ^b^
Proteins (g/dL)	7.17 ± 0.22 ^a^	6.36 ± 0.33 ^b^	5.29 ± 0.32 ^c^
Total cholesterol (mg/dL)	111 ± 8.26 ^a^	90.5 ± 5.61 ^b^	78.0 ± 8.16 ^c^
HDL-cholesterol (mg/dL)	33.9 ± 3.21	30.6 ± 2.46	31.7 ± 5.00
LDL-cholesterol (mg/dL)	13.1 ± 1.29	12.3 ± 1.78	11.8 ± 1.77
VLDL-cholesterol (mg/dL)	63.5 ± 8.94 ^a^	47.5 ± 3.51 ^b^	35.7 ± 7.36 ^c^
Triglycerides (mg/dL)	81.9 ± 12.4 ^a^	52.8 ± 14.7 ^b^	43.1 ± 9.85 ^b^
ALT (U/L)	36.8 ± 8.50 ^a^	25.4 ± 2.72 ^b^	21.6 ± 1.99 ^b^
AST (U/L)	112 ± 13.3 ^a^	69.3 ± 7.27 ^b^	58.4 ± 3.38 ^c^
Lycopene in plasma (μg/mL)	nd	0.013 ± 0.001 ^b^	0.016 ± 0.001 ^a^
ORAC in plasma (mmoles TE/L)	7.56 ± 0.33 ^c^	10.4 ± 0.27 ^b^	13.1 ± 0.74 ^a^
MDA plasma (nmol MDA/mL)	7.42 ± 0.76 ^a^	3.88 ± 0.47 ^b^	3.39 ± 0.44 ^b^
MDA liver (nmol MDA/g protein)	1.05 ± 0.14 ^a^	0.59 ± 0.16 ^b^	0.51 ± 0.08 ^b^
MDA urine (nmol MDA/mg creatinine)	14.0 ± 1.75 ^a^	6.20 ± 2.43 ^b^	2.97 ± 1.13 ^c^

^1^ Data are expressed as mean value for each group (*n* = 6 for DC group, *n* = 8 for LC12.75 and HC25.5 groups) analyzed individually in triplicate ± SD. ^a–c^ Different letters show significant statistical differences between the groups after one-way ANOVA (*p* < 0.05). CD, Standard diet; LC12.75, Standard diet + 12.75% spinach and tomato mixture; HC25.5, Standard diet + 25.5% spinach and tomato mixture.

**Table 3:** Carotenoid content (μg/g) in the liver of rats from the three experimental groups at the end of the five-week intervention period ^1^.

Carotenoids	CD	LC12.75	HC25.5
Neoxanthin	nd	5.58 ± 2.21 ^b^	10.2 ± 1.73 ^a^
Violaxanthin	nd	3.82 ± 1.54 ^b^	7.35 ± 1.46 ^a^
Lutein	nd	63.6 ± 8.86 ^b^	127 ± 19.0 ^a^
Zeaxanthin	nd	9.94 ± 1.15 ^b^	13.68 ± 1.96 ^a^
Phytoene	nd	66.8 ± 18.4 ^b^	103 ± 15.3 ^a^
α-carotene	nd	8.82 ± 2.61 ^b^	28.0 ± 5.46 ^a^
β-carotene	nd	51.2 ± 10.1 ^b^	76.3 ± 13.7 ^a^
Lycopene	nd	379 ± 108 ^b^	676 ± 64.9 ^a^
Total	nd	589 ± 142 ^b^	1043 ± 107 ^a^

^1^ Values are expressed as mean value for each group (*n* = 6 for DC group, *n* = 8 for LC12.75 and HC25.5 groups) analyzed individually in triplicate ± SD. ^a,b^ Different letters show significant statistical differences (*p* < 0.05) between the groups after one-way ANOVA. CD, Standard diet; LC12.75, Standard diet + 12.75% spinach and tomato mixture; HC25.5, Standard diet + 25.5% spinach and tomato mixture.

**Table 4:** Total fat, total cholesterol, triglycerides and fatty acid (mg/g) concentrations in the liver tissues of rats of the three experimental groups at the end of the five-week intervention period ^1^.

Parameters	CD	LC12.75	HC25.5
Total fat (%)	12.9 ± 0.12 ^a^	12.2 ± 0.35 ^a,b^	11.9 ± 0.00 ^b^
Total cholesterol (mg/g)	8.37 ± 0.65 ^a^	4.96 ± 0.47 ^b^	4.01 ± 0.44 ^c^
Triglycerides (mg/g)	13.81 ± 1.47 ^a^	9.89 ± 1.93 ^b^	7.43 ± 1.39 ^c^
Caprylic acid (C8:0)	0.013 ± 0.003 ^a^	0.011 ± 0.004 ^a^	0.006 ± 0.001 ^b^
Capric acid (C10:0)	0.033 ± 0.002 ^a^	0.012 ± 0.007 ^b^	0.007 ± 0.001 ^b^
Undecanoic acid (C11:0)	0.042 ± 0.007 ^a^	0.019 ± 0.004 ^b^	0.014 ± 0.001 ^c^
Lauric acid (C12:0)	0.024 ± 0.008 ^a^	0.014 ± 0.005 ^a^	0.007 ± 0.002 ^b^
Myristic acid (C14:0)	0.035 ± 0.003 ^a^	0.020 ± 0.006 ^b^	0.018 ± 0.002 ^b^
Pentadecanoic acid (C15:0)	0.015 ± 0.001 ^a^	0.010 ± 0.001 ^b^	0.010 ± 0.001 ^b^
Palmitic acid (C16:0)	0.038 ± 0.010 ^a^	0.020 ± 0.004 ^b^	0.016 ± 0.004 ^b^
Margaric acid (C17:0)	0.020 ± 0.003 ^a^	0.016 ± 0.002 ^b^	0.015 ± 0.001 ^b^
Stearic acid (C18:0)	0.810 ± 0.035 ^a^	0.540 ± 0.045 ^b^	0.535 ± 0.032 ^b^
Arachidic acid (C20:0)	0.046 ± 0.015 ^a^	0.026 ± 0.006 ^b^	0.019 ± 0.005 ^b^
Docosanoic acid (C22:0)	0.048 ± 0.016 ^a^	0.028 ± 0.005 ^b^	0.020 ± 0.005 ^b^
Tetracosanoic acid (C24:0)	nd	nd	0.032 ± 0.006
Myristoleic acid (C14:1)	0.052 ± 0.015 ^a^	0.022 ± 0.001 ^b^	0.017 ± 0.004 ^c^
Cis Pentadecanoic acid (C15:1)	nd	0.007 ± 0.001 ^b^	0.016 ± 0.004 ^a^
Palmitoleic acid (C16:1)	0.116 ± 0.008 ^a^	0.067 ± 0.008 ^b^	0.047 ± 0.002 ^c^
Cis Heptadecenoic acid (C17:1)	nd	nd	0.010 ± 0.004
Elaidic acid (C18:1n9t)	0.106 ± 0.014 ^b^	0.119 ± 0.010 ^b^	0.167 ± 0.041 ^a^
Oleic acid (C18:1n9c)	0.372 ± 0.078 ^c^	0.562 ± 0.090 ^b^	0.916 ± 0.067 ^a^
Eicosenoic acid (C20:1n9)	nd	nd	0.006 ± 0.001
Nervonic acid (C24:1n9)	0.036 ± 0.003 ^b^	0.051 ± 0.009 ^a^	0.057 ± 0.010 ^a^
Linolelaidic acid (C18:2tn-6)	nd	0.087 ± 0.005 ^b^	0.096 ± 0.007 ^a^
Linoleic acid (C18:2cn-6)	0.358 ± 0.040 ^c^	0.526 ± 0.062 ^b^	0.858 ± 0.021 ^a^
γ-Linolenic acid (C18:3n-6)	0.014 ± 0.001 ^a^	0.009 ± 0.001 ^b^	0.009 ± 0.002 ^b^
α-Linolenic acid (C18:3n-3)	nd	0.019 ± 0.003 ^b^	0.026 ± 0.004 ^a^
Eicosadienoic acid (C20:2n-6)	nd	nd	0.008 ± 0.001
Dihomo-γ-linolenic acid (C20:3n-6)	0.029 ± 0.006	0.022 ± 0.003	0.029 ± 0.010
Arachidonic acid (C20:4n-6)	0.401 ± 0.050 ^b^	0.540 ± 0.044 ^a^	0.556 ± 0.054 ^a^
Dihomo-α-linolenic acid (C20:3n-3)	nd	0.012 ± 0.001 ^b^	0.077 ± 0.002 ^a^
Eicosapentaenoic acid (C20:5n-3)	nd	0.023 ± 0.004 ^b^	0.026 ± 0.003 ^a^
Docosahexaenoic acid (C22:6n-3)	0.125 ± 0.004 ^c^	0.142 ± 0.017 ^b^	0.163 ± 0.014 ^a^
SAFA/TFA ratio (%)	41.1 ± 2.08 ^a^	24.49 ± 2.06 ^b^	18.50 ± 1.10 ^c^
MUFA/TFA ratio (%)	24.9 ± 2.34 ^b^	28.3 ± 3.44 ^b^	32.6 ± 2.02 ^a^
PUFA/TFA ratio (%)	34.0 ± 1.59 ^b^	47.2 ± 2.57 ^a^	48.0 ± 0.96 ^a^
n-6/n-3 ratio	6.43 ± 0.43 ^a^	6.07 ± 0.46 ^a^	5.34 ± 0.26 ^b^

^1^ Data are expressed as mean value for each group (*n* = 6 for DC group, *n* = 8 for LC12.75 and HC25.5 groups) analyzed individually in triplicate ± SD. ^a–c^ Different letters show statistically significant differences between experimental groups after one-way ANOVA (*p* < 0.05). CD, Standard diet; LC12.75, Standard diet + 12.75% spinach and tomato mixture; HC25.5, Standard diet + 25.5% spinach and tomato mixture; TFA, Total fatty acid; SAFA, Saturated fatty acid; MUFA, Monounsaturated fatty acid; PUFA, Polyunsaturated fatty acid; nd, not detected.

**Table 5:** The gene symbol, the name of the gene and the relative change of the genes that showed an expression value greater than 1.5 (*p* < 0.05) in the rat livers ^1^.

Symbol	Gene Name	Fold Change	
		CD-LC12.75	CD-HC25.5
*β-oxidation of fatty acids*			
*Acox1*	Acyl-Coenzyme A oxidase 1, palmitoyl	4.48	3.39
*Acadl*	Acyl-Coenzyme A dehydrogenase, long-chain	6.32	-
*Cholesterol transport and metabolism*			
*ApoA1*	Apolipoprotein A-1	-	4.64
*Cyp2e1*	Cytochrome P450, family 2, subfamily e, polypeptide 1	6.92	-
*Cnbp*	CCHC-type zinc finger, nucleic acid-binding protein	-	3.11
*Nr1h2*	Nuclear receptor subfamily 1, group H, member 2	-	1.75
*Ppard*	Peroxisome proliferator-activated receptor delta	-	9.55
*Other lipid transport and metabolism*			
*Acsm3*	Acyl-CoA synthetase medium-chain family member 3	3.32	-
*Hnf4a*	Hepatocyte nuclear factor 4, Alpha	4.97	-

^1^ The change in times for each gene in groups LC12.75 and HC25.5 was calculated, taking reference a value of 1 for the CD group.

## Supplementary Materials

The following are available online at https://www.mdpi.com/2076-3921/9/11/1041/s1, Figure S1. Experimental study design. Table S1. Proximal composition, energy values, phenolic compounds, antioxidant capacity and carotenoid content of the diets administered in the three experimental groups.^1^.

## Abbreviations

NF-κB	Nuclear factor kappa B
MAPK	Mitogen-activated protein kinase
HDL	High-density lipoprotein
LDL	Low-density lipoproteins
VLDL	Very low-density lipoprotein
