# Supplementary material for: Consumption of Spinach and Tomato Modifies Lipid Metabolism, Reducing Hepatic Steatosis in Rats
Source: Antioxidants (Basel). 2020 Oct 24;9(11):1041. doi: 10.3390/antiox9111041 (PMC7690917; doi:10.3390/antiox9111041)
Supplement: Supplementary file 1 [file antioxidants-09-01041-s001.pdf]

Supplementary Material

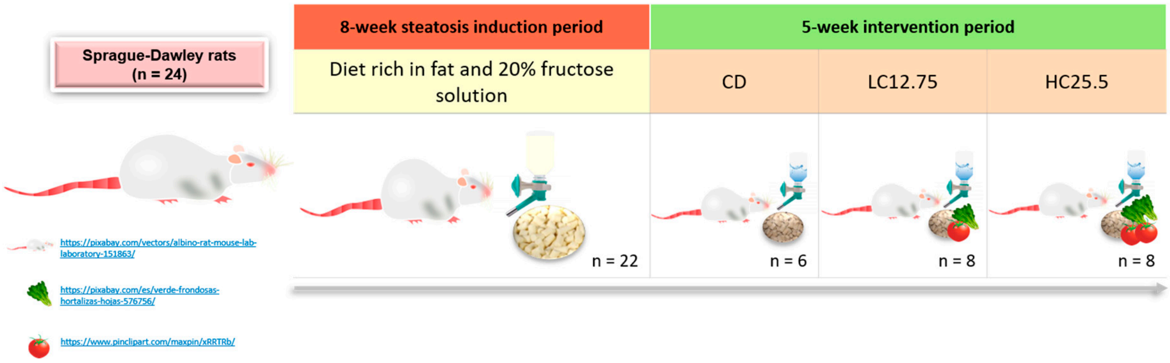

Figure S1. Experimental study design.

Table S1. Proximal composition, energy values, phenolic compounds, antioxidant capacity and carotenoid content of the diets administered in the three experimental groups.[1]

Nutrient amount (per 100 g)	CD	LC12.75	HC25.5
Protein (g)	14.33 ± 0.15 [b]	15.79 ± 0.75 [a]	16.63 ± 0.14 [a]
Fat (g)	2.91 ± 0.06 [b]	3.28 ± 0.18 [a]	3.43 ± 0.14 [a]
Total dietary fiber (TDF) (g)	21.16 ± 1.73 [b]	21.27 ± 0.9 [b]	25.63 ± 1.01 [a]
Ash (g)	1.51 ± 0.16 [c]	1.85 ± 0.11[b]	2.45 ± 0.03 [a]
Carbohydrate (g)	60.09 ± 1.48 [a]	57.81 ± 1.83 [a]	51.85 ± 0.99 [b]
Energetic value (kcal)	323.9 ± 6.09 [a]	323.93 ± 2.84 [a]	306.59 ± 2.73 [b]
Calories from protein (%)	17.7 ± 0.15 [b]	19.5 ± 1.08 [b]	21.68 ± 0.05 [a]
Calories from fat (%)	8.1 ± 0.29 [b]	9.12 ± 0.59 [ab]	10.14 ± 0.38 [a]
Calories from carbohydrate (%)	74.2 ± 0.44	71.38 ± 1.63	68.26 ± 0.45
Total phenolic (TPC) (mg GAE)	205.03 ± 1.71[c]	243.66 ± 4.47 [b]	265.30 ± 5.69 [a]
ORAC (mmoles TE)	4.59 ± 0.35 [c]	5.59 ± 0.34 [b]	8.98 ± 0.33 [a]
**Carotenoids (µg/100 g)**			
Neoxanthin	-	5.64 ± 1.11 [b]	8.36 ± 0.34 [a]
Violaxanthin	-	3.24 ± 0.91 [b]	5.13 ± 0.64 [a]
Lutein	-	58.71 ± 2.39 [b]	95.05 ± 4.51 [a]
Zeaxanthin	-	4.68 ± 0.62 [b]	9.15 ± 1.27 [a]
Phytoene	-	44.02 ± 8.86 [b]	84.69 ± 7.18 [a]
α-carotene	-	8.51 ± 0.74 [b]	18.33 ± 1.05 [a]
β-carotene	-	46.76 ± 2.00 [b]	91.96 ± 1.17 [a]
Lycopene	-	113.28 ± 17.40 [b]	218.77 ± 17.28 [a]
Total	-	283.84 ± 8.46 [b]	528.65 ± 24.29 [a]

[1] Values are expressed as mean ± SD. [a–c] Different letters in the same row show significant statistically differences between groups after performing a one-way ANOVA (p < 0.05). CD: Standard diet (Teklad Global 14% Protein Rodent Maintenance Diet, 2014), LC12.75: Standard diet + 12.75% spinach and tomato mixture, HC25.5: Standard diet + 25.5% spinach and tomato mix.
